# Association of pulmonary embolism and acute coronary syndrome during COVID-19 infection: Case report and a brief review

**DOI:** 10.1016/j.amsu.2021.103152

**Published:** 2021-12-04

**Authors:** Abdelaziz boudihi, Charmake Derar, Mosaab Mazouzi, Nabila ismaili, Noha el ouafi

**Affiliations:** a Faculty of Medicine and Pharmacy, Mohammed I^st^ University, Oujda, Morocco; b Department of Cardiology, Mohammed VI University Hospital Mohammed I University, Oujda, Morocco

**Keywords:** Acute coronary syndrome, Pulmonary embolism, COVID-19-Anticoagulant-Thrombo-embolic event

## Abstract

**Introduction and importance:**

COVID 19 infection is considered a potentially serious disease since it is responsible for important respiratory and cardiovascular complications with a high morbid-mortality.

**Case presentation:**

We report the case of a 54-year-old diabetic patient with hypertension who was admitted for heart failure with a reduced LVEF of 23% triggered by a pulmonary embolism and an acute coronary syndrome in the context of COVID-19 infection.

**Clinical discussion:**

Indeed, these complications may be secondary to a prothrombotic and hypercoagulable state as well as endothelial dysfunction caused by the vascular and systemic inflammation and cytokine storm induced by SARS-CoV-2. Although the clinical polymorphism of COVID 19 infection is recognized, the association of myocardial ischemia with pulmonary embolism is uncommon and of adverse prognosis. This justifies a rapid and adapted multidisciplinary management.

**Conclusion:**

In the absence of contraindication, thromboprohylaxis should be initiated for all hospitalized patients with COVID-19 to minimize the risk of thromboembolic complications.

## Highlights

• Covid-19 should be considered as a high risk thrombogenic disease
• Endothelial dysfunction during the inflammatory storm plays an important role in thromboembolic events
• Association of pulmonary embolism and acute coronary syndrome is rare in the context of COVID-19 infection
• Curative anticoagulation should be discussed in all patients hospitalized with covid-19 infection

## 1 Introduction

Since its emergence in China, the increased risk of thrombosis, either venous or arterial, associated with COVID19 infection has been demonstrated by several studies. Because of their high incidence, especially in cases of severe disease, and their considerable mortality rate [1], these complications remain a major medical challenge, both in terms of diagnosis and prognosis, despite well-managed preventive anticoagulation. Here we will describe the case of a patient with COVID-19 infection concomitantly complicated by pulmonary embolism and acute coronary syndrome. From this case we want to show one of the serious situations that can occur during this pandemic such as the coincidence between an acute coronary syndrome and a pulmonary embolism and the therapeutic guidelines that can be followed.

## 2 Case presentation

We report the case of a 54-year-old patient, with no previous pathological history, who initially consulted for a symptomatology consisting of dyspnea, asthenia, and myalgia associated with an lower limbs edema since two months.

On admission, the patient was in congestive heart failure: dyspnea, blood pressure (BP) was 140/70 mmHg, heart rate (HR) was 110 bpm, spontaneous SpO2 was 88%, and he had basithoracic crepitus rales and lower extremity edema.

**Fig. 1:**
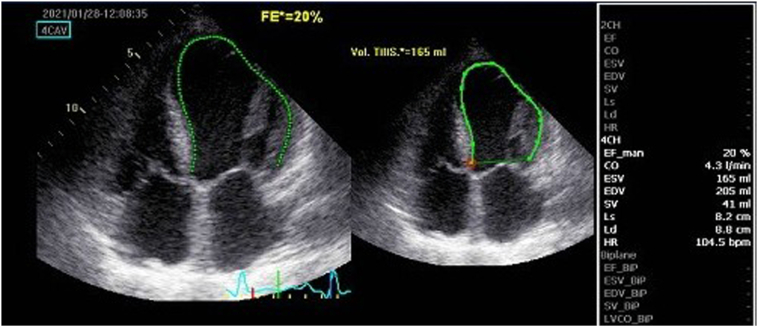
Apical view of TTE that shows a severe left ventricular dysfunction in simpson biplane.

**Fig. 2:**
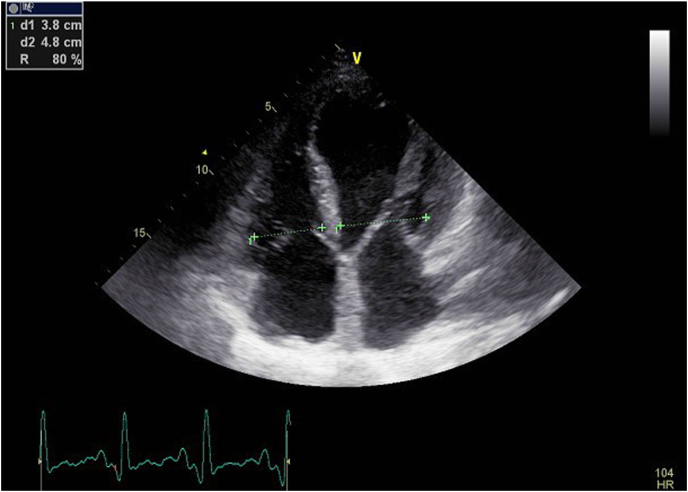
Apical view of TTE that shows a dilated right ventricle.

The electrocardiogram showed sinus rhythm with left ventricular hypertrophy with repolarization abnormalities (anterior Q waves and high lateral negative T waves). Transthoracic echocardiography (TTE) revealed a pattern of dilated cardiomyopathy (DCM) with severe biventricular dysfunction (left ventricular ejection fraction (LVEF): 23%, dilated right ventricle with limited systolic function), associated with left ventricular wall motion abnormalities, spontaneous contrast image, increased left ventricular filling pressures with an elevated PAPS at 60 mmHg (Fig. 1, Fig. 2).

**Fig. 3:**
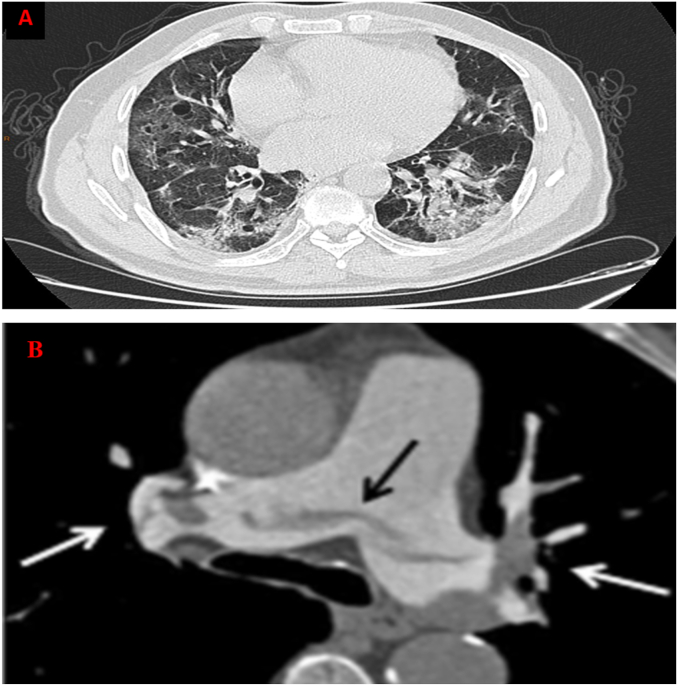
**A)** thoracic CT scan showing diffuse patchy ground-glass opacities suggestingCOVID-19 pneumonia, **B)** a chest CT scan with injection showing bilateral proximal pulmonary embolism.

Biologically, we notice an inflammatory syndrome with C-reactive protein at 275 mg/l (Normal Value 6–12mg/l) and WBC at 16530/mm3, (Normal value 4000–10000/mm3), The ultra-sensitive troponin is positive with a first determination at 7249 pg/ml and the second at 8579 pg/ml (Normal value 0–26 pg/ml).The renal function test, blood electrolytes and hemoglobin level were normal. In front of this endemic context, a first polymerase chain reaction (PCR) test on nasopharyngeal swab was performed with a positive result. A thoracic CT scan without and with injection showed a worsening of the lesions, with an estimated damage of more than 25%–50% with a bilateral pulmonary embolism (Fig. 3)

**Fig. 4:**
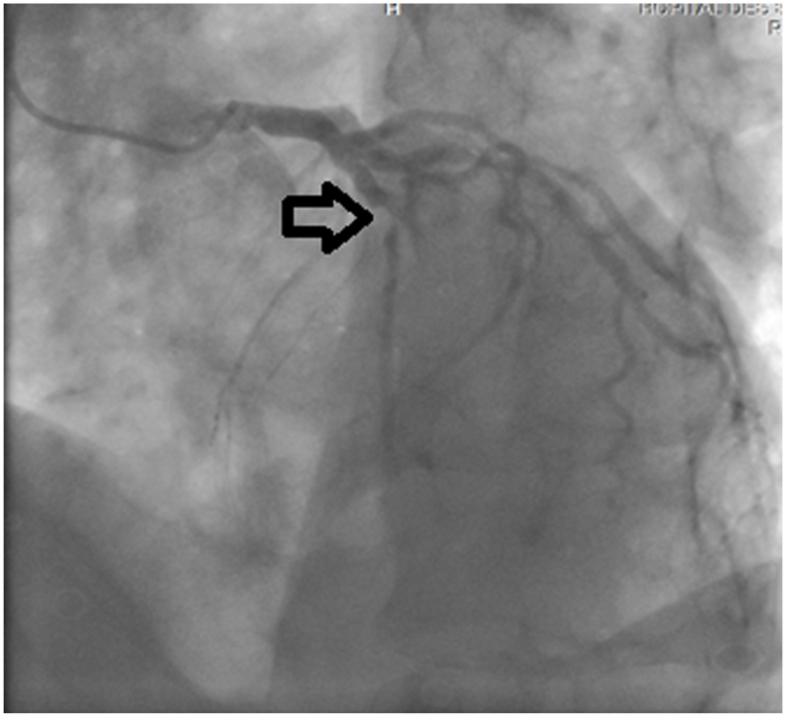
Coronary image showing a thrombus in a coronary artery.

In view of a positive troponin cycle and abnormalities of wall motion on TTE, coronary angiography is performed revealed a tri-truncular involvement (sub occlusive stenosis of the middle LAD, tight stenosis of the middle and distal proximal marginal and tight stenosis of the first segment of the RCA) with multiple thrombi (Fig. 4)

For therapeutic management, our patient had received oxygen therapy, injectable diuretic therapy and conventional Heart failure reducted ejection fraction (HFrEF) treatments including beta blocker, ACEi and spironolactone associated with dual antiplatelet therapy and anticoagulation initially parenteral with enoxaparin then oral with acenocoumarol, associated with the national anti-covid protocol based on Zinc 45mg/d, Vit C 1000mg/D and corticosteroid therapy by dexamethasone 6mg/day given the marked inflammatory state.

The evolution under treatment was favorable with regression of the signs of heart failure and good tolerance to the introduced treatments. The patient is transferred at day 5 of his admission for a possible coronary bypass with a good clinical improvement.

## 3 Discussion

Since its declaration on March 11, 2020, as a pandemic by the World Health Organization [2], the association between adverse cardiovascular events, such as acute myocardial infarction and pulmonary embolism, and COVID-19 has been demonstrated in several studies.

In addition to the cytokine storm and endothelial injury [3,[4], [22]], several factors are responsible for the hypercoagulable state frequently associated with covid 19 infection including the sepsis state, inflammation, hypoxia, immobilization and diffuse intravascular coagulation responsible for the release of lactate dehydrogenase (LDH), ferritin, C-reactive protein, D-dimers and interleukin into the systemic circulation [5,6]. Indeed, the inflammatory state generated by this infectious process could activate the inflammatory cells constituting atherosclerotic plaques [7].

Furthermore, signaling pathways related to angiotensin-converting enzyme 2 (ACE2) receptors may also play a role in the genesis of myocardial injury [8]. In fact, these receptors are the entry point of some coronaviruses such as SARS-CoV2 into human cells especially in the lungs and cardiovascular system where they are highly expressed [9].

The epidemic context associated with Covid 19 makes the confirmation of the diagnosis of acute coronary syndromes, especially Non-ST-Elevation Myocardial Infarction (NSTEMI), more difficult in many cases because of the unreliability of troponin elevation, which can be elevated in many contexts, and the underutilization of coronary angiography given the infectious risk [8]. However, troponin levels may reflect the severity of SARS-CoV2 infection. In fact in a meta-analysis by Lippi et al. severe SARS-CoV-2 infection was associated with higher troponin levels compared to those found in mild forms of the disease [10].

In the therapeutic management of patients admitted with acute coronary syndrome concomitant with SARS-CoV-2 infection, the risk of infection should be considered without delaying anti-ischemic management, according to many authors [11,12] thrombolytic therapy was recommended in STEMI rather than primary PCI if Covid-19 was confirmed or could not be excluded quickly, whereas for NSTEMI, the priority was to exclude SARS-CoV-2 infection first. These recommendations were endorsed by Daniels et al. According to this study, thrombolysis may be the best compromise for rapid patient reperfusion to gain time to treat and establish a complete diagnosis [13].

Yet, according to several guidelines from the American College of Cardiology, the American College of Emergency Physicians (ACEP) and the American Heart Association [14,15] primary angioplasty should remain the first choice and should be performed within an adequate time frame after the onset of symptoms, whenever possible, after an initial assessment in the emergency department of the risk of infection and thrombolytic therapy should not be the standard of care strategy and should be limited to specific situations.

In the same sense, the European and American general guidelines on STEMI [16,17], confirm that primary PCI remains the reperfusion treatment of choice if it is feasible within an acceptable time frame after the diagnosis of STEMI.

The platelet hyperactivity that accompanies COVID-19 infection shown in several studies, makes antiplatelet therapy very useful [18], especially in ischemic heart disease like our case. In addition, the use of aspirin and other non-steroidal anti-inflammatory drugs (NSAIDs) does not have serious adverse effects in patients with COVID-19, as stated by the WHO [19].

For thromboembolic risk assessment, it is recommended that the severity of COVID-19 be taken into consideration, based on the type of oxygen therapy required for the patient (none, nasal oxygen therapy, high-flow oxygen therapy, or mechanical ventilation) and the body mass index (BMI).

Some authors [20] propose to classify patients according to their thrombotic risk as follows:

- Low risk: non-hospitalized patients with a body mass index (BMI) < 30 kg/m^2^, without associated risk factors.
- Intermediate risk: patients with BMI <30 kg/m^2^, with or without associated risk factors, without the need for high-flow nasal oxygen therapy (HFO) or artificial ventilation.
- High risk: BMI <30 kg/m^2^, with or without associated FDR, on HFO or artificial ventilation; BMI >30 kg/m^2^ without associated risk factors; BMI >30 kg/m2 with associated risk factors, on HFO or artificial ventilation.
- Very high risk: BMI >30 kg/m2 with risk factors, on HFO or artificial ventilation.

In any case, prophylactic anticoagulation is recommended for all hospitalized COVID-19 patients, and only during the hospitalization period.These same authors propose a dose of enoxaparin 4,000 IU/24h subcutaneous (SC) for intermediate-risk patients, a dose of enoxaparin 4,000 IU/12h SC for high-risk patients, and the use of curative doses of enoxaparin 100 IU/kg/12h SC, for very high-risk patients.

In cases of renal impairment (creatinine clearance <30 ml/min), unfractionated heparin (UFH) is proposed at a dose of 200 IU/kg/24h for high risk and 500 IU/kg/24h for very high risk. It is also recommended that enoxaparin doses be adjusted for patients weighing >120 kg and that anti-Xa activity be measured regularly [20].According to some authors [20], direct oral anticoagulants (DOAs) are a good alternative for patients without contraindications or drug interactions. For critical patients in the ICU setting with pulmonary embolism or DVT, LMWH or fondaparinux are preferred to DOAs. The period of anticoagulation should be three months. If there is a recurrence of thromboembolic events on DOAs, it is necessary to switch back to LMWH [20].

The SCARE guidelines were used in the writing of this paper [21].

## 4 Conclusion

SARS-CoV-2 infection has been associated with major cardiovascular complications, including acute myocardial infarction, myocarditis, heart failure, arrhythmia, and venous thromboembolic disease. In the absence of contraindication, thromboprohylaxis should be initiated for all hospitalized patients with COVID-19 to minimize the risk of thromboembolic complications.

## Funding

None.

## Ethical approval

The ethical committee approval was not required give the article type (case report).However, the written consent to publish the clinical data of the patients was given and is available to check by the handling editor if needed.

## Sources of funding

This research did not receive any specific grant from funding agencies in the public, commercial, or not-for-profit sectors.

## Author contribution

Abdelaziz Boudihi : study concept or design, data collection, data analysis or interpretation, writing the paper.

Charmake Derare : data collection and management.

Mossab mazouzi : data collection.

Nabila ismaili : supervision and data validation.

Noha El ouafi : supervision and data validation.

## Consent

Written informed consent was obtained from the patient for publication of this case report and accompanying images. A copy of the written consent is available for review by the Editor-in-Chief of this journal on request.

## Registration of Research Studies

This is not an original research project involving human participants in an interventional or an observational study but a case report . This registration is was not required.

## Guarantor

ABDELAZIZ BOUDIHI.

## Declaration of competing interest

The authors state that they have no conflicts of interest for this report Ethical Approval The ethical committee approval was not required give the article type case report. However, the written consent to publish the clinical data of the patients were given and is available to check by the handling editor if needed**.**
